# Supplementary material for: Evaluating dyadic factors associated with self-care in patients with heart failure and their family caregivers: Using an Actor-Partner Interdependence Model
Source: PLoS One. 2024 Sep 19;19(9):e0308515. doi: 10.1371/journal.pone.0308515 (PMC11412497; doi:10.1371/journal.pone.0308515)
Supplement: Attachment — Submitted filename: R1_PlosOne_response-to-reviewers.doc [file pone.0308515.s002.doc]

February 22, 2024

Shigao Huang

Academic Editor

Plos One

Dear Editor:

We would like to thank you for your thoughtful comments and recommendations. We have made the following changes to the manuscript to address your concerns and revised the paper, paying particular attention to language. We obtained additional editing support from a scientific editing company. The revised sections of the manuscript are indicated in RED.

| Reviewer #1 Comments | Authors’ Responses |
| --- | --- |
| In this article entitled “Evaluating dyadic factors associated with self-care in patients with heart failure and their family caregivers: using an Actor-Partner Interdependence Model” the authors analyze how relationships of patient-caregiver physical function and depressive symptoms to the self-care behavior of the disease. A study highlighting the need to assess the physical and psychological conditions of both patients and caregivers, and the importance of caregiver assistance in self-care. The manuscript addresses an important and interesting topic, especially that chronic HF is increasing in prevalence with an ageing population. However, I have a few comments on some issues that require more attention. | Thank you for taking the time to review our manuscript and for providing valuable feedback. We are pleased to hear that you find the topic of our manuscript important and interesting, particularly in the context of the increasing prevalence of chronic heart failure with an aging population. We have made the following changes to the manuscript to address your concerns below. |
| Introduction: • In addition to the recent review mentioned in the authors' review, I found a more recent review: Uchmanowicz I, et al. Heart Failure Care: Testing Dyadic Dynamics Using the Actor-Partner Interdependence Model (APIM)-A Scoping Review. Int J Environ Res Public Health. 2022 Feb 9;19(4):1919. doi: 10.3390/ijerph19041919. PMID: 35206131; PMCID: PMC8871794, and it seems reasonable to include it as well, particularly as it talks about the emotional components of HF management within the framework of APIM. | We appreciate your effort in finding additional relevant literature. In response to your suggestions, we have incorporated this information into the Introduction section (Page 4) as follows:  In patients with HF, depressive symptoms were consistently associated with poor self-care in a systematic literature review [30] and in caregivers of HF patients, the absence of depressive symptoms or anxiety was associated with an improved quality of life for the patients [31]. |
| Discussion • the discussion part could be a little bit shorter and easier to follow. | As per your suggestion, we carefully reviewed the section and work towards making it more succinct and easier to follow without compromising the depth of the content (Pages 17-23). |
| • In the discussion section, I would recommend avoiding figures from other authors' studies such as," In China, the mean scores of CCPS maintenance and management were 52.4 and 55.6 [63], which are similar or somewhat lower than those in Italy." | As per your suggestion, we deleted the figures from other studies and revised the discussion section accordingly (Page 19).  In China [63], the mean scores of CCPS maintenance and management were similar or somewhat lower than those in Italy. |

| Reviewer #2 Comments | Authors’ Responses |
| --- | --- |
| This study used a cross-sectional, correlational design to investigate the physical function and depressive symptom status of patients with heart failure (HF) and their caregivers as dyads, examining their associations with patient self-care and caregiver contribution to patient self-care (CCPS).  The study collected data from four university-affiliated hospitals in South Korea between June 12, 2019, and October 29, 2021.  The study focused on patient-caregiver dyads, which provides a more comprehensive understanding of the interactions between patients and their caregivers, as opposed to solely studying patients or caregivers in isolation.  Heart failure is a significant health concern, and understanding the factors affecting self-care and caregiver contributions is essential for improving the quality of care for patients with HF.  The study employed well-established tools, such as the Revised Self-Care of Heart Failure Index and the Patient Health Questionnaire-9, to assess self-care and depressive symptoms. This enhances the validity of the findings.  The study was conducted following ethical guidelines, including obtaining informed consent from participants and approval from Institutional Review Boards, ensuring the ethical conduct of research. | Thank you for your thoughtful and positive comments on our study. We are grateful for your recognition of the strengths and rigor of our research. |
| The study used a convenience sampling method, which may limit the generalizability of the findings to a broader population. Convenience samples might not represent the entire population of patients with HF and their caregivers. | We appreciate this insightful comment. To address the limitation associated with our use of convenience sampling, we have expanded the discussion on this aspect in the future study section (Page 23):  Patients and their caregivers were recruited using a convenience sampling method, limiting the generalization of the study findings.  The study findings propose several approaches for future research. Firstly, exploring larger samples with random sampling can enhance the robustness of conclusions. |
| The study's cross-sectional design limits its ability to establish causal relationships. Longitudinal studies would be more suitable for examining changes over time and identifying causality. You should addresses it in the paper as the study limitations. | We have incorporated this concern into the limitation section and expanded the discussion on this aspect in the future study section (Page 23):  Further, the nature of the cross-sectional design in this study remains unknown whether improvements in physical and psychological conditions of patient-caregiver dyads will lead to improvements in the dyadic patient self-care and CCPS.  Secondly, employing a longitudinal design to examine changes over time and establish causality is recommended. |
| While the sample size was deemed adequate for the power analysis, larger samples could enhance the robustness of the findings, especially given the complexity of the dyadic relationships being examined. | We appreciate this insightful comment. To address the limitation associated with sample size, we have expanded the discussion on this aspect in the future study section (Page 23):  The study findings propose several approaches for future research. Firstly, exploring larger samples with random sampling can enhance the robustness of conclusions. |
| The study was conducted in South Korea, which may have cultural and regional nuances affecting self-care and caregiver contributions. The findings may not be directly transferable to other cultural or geographical contexts. | This is an excellent point. We expanded the study limitation to discuss this (Page 22):  Moreover, the study findings may be influenced by cultural factors impacting patient self-care and CCPS, limiting their direct applicability to other cultural or geographical contexts. |
| The study primarily focused on physical function and depressive symptoms as factors influencing self-care. Other variables, such as socioeconomic status, education, and social support, may also play significant roles but were not explored. | As suggested, we have included recommendations for future research that could explore the impact of additional variables on self-care in greater detail as follows (Page 23):  Thirdly, considering the incorporation of various characteristics, including socioeconomic status, education, and social support, can contribute to gaining a more comprehensive understanding of factors associated with self-care. |
| In summary, this study offers valuable insights into the relationships between physical function, depressive symptoms, self-care, and caregiver contributions in patient-caregiver dyads dealing with heart failure. However, it has limitations, and future research should aim for larger, more diverse samples and consider additional factors that may impact self-care and caregiving. | We appreciate this insight comment. As suggested, we have expanded our discussion to address the limitation and future research approaches (Page 23). |

Thank you again for your constructive feedback.

Sincerely,

Authors
