# Supplementary material for: Evaluating dyadic factors associated with self-care in patients with heart failure and their family caregivers: Using an Actor-Partner Interdependence Model
Source: PLoS One. 2024 Sep 19;19(9):e0308515. doi: 10.1371/journal.pone.0308515 (PMC11412497; doi:10.1371/journal.pone.0308515)
Supplement: Attachment — Submitted filename: R2_PlosOne_response-to-reviewers_07012024.doc [file pone.0308515.s003.doc]

July 22, 2024

Shigao Huang

Academic Editor

Plos One

Dear Editor:

Thank you very much for your feedback regarding the reference list in our manuscript titled “Evaluating Dyadic Factors Associated with Self-Care in Patients with Heart Failure and Their Family Caregivers: Using an Actor-Partner Interdependence Model.” We have thoroughly reviewed our references and incorporated the necessary adjustments. The revised sections of the manuscript are indicated in RED.

| Journal Requirements | Authors’ Responses |
| --- | --- |
| Please review your reference list to ensure that it is complete and correct. If you have cited papers that have been retracted, please include the rationale for doing so in the manuscript text, or remove these references and replace them with relevant current references. Any changes to the reference list should be mentioned in the rebuttal letter that accompanies your revised manuscript. If you need to cite a retracted article, indicate the article’s retracted status in the References list and also include a citation and full reference for the retraction notice. | Thank you for taking the time to review our manuscript and for providing valuable feedback. We reviewed our reference list to ensure all entries are complete and correct. We have identified the papers in our reference list and we believe these references are still relevant. We have clearly indicated their status in the References list. We just made the adjustments based on Vancouver style format. We have detailed all changes to the reference list in the revised manuscript. |

| Reviewer #2 Comments | Authors’ Responses |
| --- | --- |
| Since the title is built on this manuscript it is worth to add the citation Uchmanowicz I, Faulkner KM, Vellone E, Siennicka A, Szczepanowski R, Olchowska-Kotala A. Heart Failure Care: Testing Dyadic Dynamics Using the Actor-Partner Interdependence Model (APIM)-A Scoping Review. Int J Environ Res Public Health. 2022 Feb 9;19(4):1919. doi: 10.3390/ijerph19041919. PMID: 35206131; PMCID: PMC8871794. | Thank you for your suggestion. We have added the citation to the introduction of our manuscript (Page 5). |

Thank you again for your constructive feedback.

Sincerely,

Authors
